# MRI patterns of non-enhancing T2-FLAIR hyperintense lesions with dynamic change in primary CNS lymphoma

**DOI:** 10.3389/fonc.2026.1718690

**Published:** 2026-07-03

**Authors:** Haiman Hou, Hao Liu

**Affiliations:** 1 Department of Neurology, The First Affiliated Hospital of Zhengzhou University, Zhengzhou, Henan, China; 2 Department of MRI, The First Affiliated Hospital of Zhengzhou University, Zhengzhou, Henan, China

**Keywords:** dynamic change, magnetic resonance imaging, non-enhancing T2-FLAIR hyperintense lesions, primary central nervous system lymphoma, T2- FLAIR sequences

## Abstract

**Purpose:**

The nature of non-enhancing T2-FLAIR hyperintense lesions that are not contiguous with the enhancing tumor site at baseline in primary central nervous system lymphoma (PCNSL) remains unclear. Our aim was to explore the incidence, location, morphology, and dynamic changes of these non-enhancing T2-FLAIR hyperintense lesions in PCNSL.

**Methods:**

We retrospectively reviewed patients diagnosed with immunocompetent PCNSL at our institution. We identified and evaluated T2-FLAIR hyperintense lesions without enhancement that showed a marked decrease or complete disappearance on MRI after treatment. MRI characteristics of PCNSL at initial presentation were analyzed and compared between patients with and without non-enhancing T2-FLAIR hyperintense lesions.

**Results:**

Among 87 patients, 10 patients (11.5%) were found to have non-enhancing T2-FLAIR hyperintense lesions located at a distance from the enhancing tumor site at baseline that showed a marked decrease or disappearance after treatment. The locations of these lesions were as follows: the juxtacortical and deep white matter (7 lesions), periventricular white matter (2 lesions), basal ganglia (1 lesion), and infratentorial area (1 lesion). Baseline MRI characteristics in patients with non-enhancing T2-FLAIR hyperintense lesions exhibited a higher rate of multiple enhancing lesions (P = 0.027) and bilateral enhancing lesions (P = 0.011) compared to patients without non-enhancing T2-FLAIR hyperintense lesions.

**Conclusion:**

In PCNSL patients, non-enhancing T2-FLAIR hyperintense lesions at a distance from enhancing tumor lesions, which are potentially neoplastic in nature, were not uncommon. Our findings raise the question of whether these non-enhancing lesions should be incorporated into the initial evaluation of tumor burden. This issue warrants further investigation.

## Introduction

Primary central nervous system lymphoma (PCNSL) is mainly composed of diffuse large B-cell lymphoma (DLBCL) that is restricted to the brain, spinal cord, leptomeninges, and eye at the time of diagnosis (1, 2). It accounts for 2.4%–4.0% of all intracranial malignant tumors and for less than 1% of all lymphomas (3–5). Treatment of PCNSL has advanced significantly, with improved survival for this rare and aggressive lymphoma of the central nervous system (CNS) after high-dose methotrexate-based chemotherapy. However, it still has a high recurrence rate, and the 5-year survival rate remains low (6).

Magnetic resonance imaging (MRI) with contrast enhancement is the most sensitive imaging modality when PCNSL is suspected. Typical MRI features of PCNSL in immunocompetent patients include single or multiple intensely homogeneously contrast-enhancing lesions with well-defined borders, which are usually localized in the periventricular areas (7–9). However, atypical radiological findings are observed in approximately 25% of PCNSL patients and may include lesions with central non-enhancing regions or lesions with susceptibility (10). Non-enhancing PCNSL lesions have also been reported at diagnosis or recurrence (11–16). DeAngelis et al. first reported 10 patients with pathologically confirmed PCNSL who demonstrated non-enhancing lesions on computed tomography or MRI. Notably, non-enhancing lesions represented the only radiographic abnormality in 5 of these 10 patients, without any enhancing lesion (17). Tabouret et al. described another interesting phenomenon in which some non-enhancing T2-FLAIR hyperintense lesions distant from the enhancing tumor site in PCNSL patients showed a marked decrease after chemotherapy (18). Furthermore, 27.8% of these patients relapsed in the initially non-enhancing T2-FLAIR lesions. This dynamic change of non-enhancing T2-FLAIR hyperintense lesions probably indicated the neoplastic nature of these lesions. Therefore, the authors proposed that the dynamic change in these non-enhancing T2-FLAIR hyperintense lesions strongly supports their neoplastic nature, and it is reasonable to incorporate these lesions into refined criteria defining response and progression in PCNSL. At our center, we have similarly observed that non-enhancing T2-FLAIR hyperintense lesions located within the white matter and distant from enhancing tumor sites in PCNSL patients markedly reduced or disappeared after chemotherapy during follow-up. However, few studies have focused on this phenomenon, and little is known about the characteristics and nature of these non-enhancing T2-FLAIR lesions that respond to chemotherapy.

Therefore, we conducted the current study to investigate the incidence, location, morphology, and dynamic changes of non-enhancing T2-FLAIR hyperintense lesions that respond to chemotherapy in PCNSL patients.

## Materials and methods

### Patients

We retrospectively reviewed consecutive immunocompetent patients with newly diagnosed PCNSL between February 2009 and September 2018 at our institution.

The eligibility criteria for this study were as follows: (1) complete and available medical records; (2) histologically confirmed diffuse large B-cell lymphoma confined to the brain at initial diagnosis, without evidence of non-CNS involvement; (3) availability of MRI images including at least T2-FLAIR, T2WI, T1WI, and T1WI with contrast-enhancement at baseline and follow-up; (4) age over 18 years old; and (5) no corticosteroid treatment before baseline MRI. Patients with poor image quality were excluded. The study was approved by the Ethics Committee of the First Affiliated Hospital of Zhengzhou University (No. 2019-KY-231). Informed consent was obtained from all individual participants included in the study.

### Neuroimaging assessment

MRI evaluations were performed at baseline, before histological diagnosis and initiation of corticosteroid treatment, and at the time of follow-up 3–6 months after treatment. All MRI scans were obtained using a 3.0-T MRI scanner (Skyra, Siemens Healthcare, German; Discovery 750, GE Medical Systems, United States). MR images, including T2WI, T2-FLAIR, T1WI, diffusion-weighted imaging (DWI), and contrast-enhanced T1-weighted imaging (CE-T1WI), were retrieved from the hospital Picture Archiving and Communication System. The inversion time for T2-FLAIR was set to 2400–2500 ms. DWI was performed using two different b-values (0 and 1000 s/mm^2^) and in three orthogonal diffusion directions. The axial CE-T1WI sequence was acquired by repeating the aforementioned T1WI after a bolus injection of 0.1 mmol/kg of gadodiamide. All MRI images were reviewed by two radiologists blinded to individual patient outcomes. Disagreements were resolved by consensus. Interobserver agreement statistics were not assessed.

### MRI analysis of non-enhancing T2-FLAIR hyperintense lesions

Initially, white matter hyperintensities on axial T2−FLAIR images of cerebral MRI were evaluated in all patients at baseline and follow-up. Then, we identified the white matter hyperintense lesions without enhancement that markedly decreased or disappeared on the follow-up MRI. The characteristics of these lesions, including number, lateralization, anatomical location, shape, signal intensity, and volume, were recorded. The location of lesions was categorized as periventricular white matter, deep white matter, juxtacortical white matter, internal capsule, and infratentorial white matter. Infratentorial localization was defined as lesions in the brainstem and/or the cerebellum. For the volume assessment, the largest diameter and the product of the largest perpendicular diameters were measured, and lesion volume was calculated using the ellipsoid volume formula. Apparent diffusion coefficient (ADC) values were measured on the axial slice showing the largest diameter of the non-enhancing T2-FLAIR hyperintense lesion. A freehand region of interest (ROI) was placed over the lesion, and the mean ADC value within the ROI was recorded. Restricted diffusion was defined as hyperintensity on DWI with corresponding hypointensity on ADC maps, and quantitatively defined as an ADC value below 0.8 × 10^-^³ mm²/s.

### MRI analysis of enhancing tumor lesions in PCNSL

For the enhancing PCNSL lesions, characteristics including number, lateralization, anatomical location, and contrast enhancement pattern of the tumor lesions at initial presentation on MRI were analyzed and documented. Lateralization was based on the Talairach atlas; deep lesions were defined as lesions located in the periventricular regions, basal ganglia, brainstem, and/or cerebellum (19). Infratentorial localization was defined as lesions in the brainstem and/or the cerebellum. Patients with more than two tumor lesions were classified as having a diffuse lesion; otherwise, lesions were classified as non-diffuse. Enhancement patterns were categorized as homogeneous or heterogeneous enhancement. Baseline MRI characteristics of PCNSL patients at initial presentation were compared between patients with and without non-enhancing T2-FLAIR hyperintense lesions.

### Statistical analysis

Clinical characteristics and imaging features are reported as means ± standard deviations or medians with ranges for continuous variables. Categorical variables are reported as frequencies or percentages. The independent-samples Mann–Whitney U test was used for between-group comparisons of continuous variables. Fisher’s exact test was used to compare dichotomous variables. A two-sided P value < 0.05 was considered statistically significant. All statistical analyses were performed using SPSS software (Version 25.0; IBM Corp., Armonk, NY, USA).

## Results

### Patient characteristics and baseline MRI characteristics

At the time of analysis, a total of 87 patients were included in the neuroimaging study. The median age of the included patients was 54 years (range: 16–80 years), and 28 (32.2%) were male. All cases were histologically confirmed as diffuse large B-cell lymphoma and confined to the brain at initial diagnosis. All patients received a uniform standard first-line multi-agent chemotherapy regimen based on high-dose methotrexate. Demographic and baseline MRI characteristics of patients are detailed in **Table 1**.

**Table 1:** Demographic and baseline MRI characteristics of the study population.

Clinical and baseline neuroimaging characteristics	Years/cases
Number of cases	87
Age (years)	54.5 ± 12.4
Male	28 (32.2%)
Number of enhancing lesion (s)	
1	50 (57.5%)
≥2	37 (42.5%)
Lesion lateralization	
Left	26 (29.9%)
Right	28 (32.2%)
Bilateral	22 (25.3%)
Midline	11 (12.6%)
Lesion location	
Cerebral hemisphere	61 (70.1%)
Basal ganglia and thalamus	29 (33.3%)
Brainstem	8 (9.2%)
Cerebellum	12 (13.8%)
Corpus callosum	15 (17.2%)
Ventricular system	4 (4.6%)
Spinal cord	1 (1.1%)
Deep^#^	62 (71.3%)
Superficial	15 (17.2%)
Deep and superficial	10 (11.5%)
Infratentorial lesions	18 (20.7%)
Brainstem	8 (9.2%)
Homogeneous	70 (80.5%)
Heterogeneous	17 (19.5%)

^#^ Deep lesions include those involving the corpus callosum, basal ganglia, brainstem, and/or cerebellum.

All 87 patients had contrast-enhancing tumor lesions on baseline MRI. A total of 50 patients (57.5%) exhibited a solitary enhancing lesion. Among the remaining 37 patients (42.5%) with multifocal lesions, the majority had more than two lesions (n = 25, 67.6%). Regarding the lateralization of the lesions, 26 cases were located on the left, 28 cases were located on the right, 22 cases were bilaterally involved, and 11 cases were located in the midline areas. In general, the majority of patients (n = 72, 82.8%) showed deeply located contrast-enhancing lesions, and 10 of them also had superficially located lesions. The most frequently involved area was the cerebral hemispheres (n = 61, 70.1%), followed by the basal ganglia and thalamus (n = 29, 33.3%), and the corpus callosum (n = 15, 17.24%). Infratentorial involvement was observed in 18 patients (20.7%), including 10 cases involving lesions in the cerebellum, 6 cases in the brainstem, and 2 cases in both the cerebellum and brainstem. Other rare locations included the ventricular system (n = 4) and spinal cord (n = 1). Supratentorial and infratentorial areas were simultaneously involved in 8 patients (9.2%). The majority of patients (n = 70, 80.5%) exhibited typical homogeneous contrast-enhancing lesions, whereas heterogeneous enhancement was observed in 19.5% of patients. Furthermore, 13 patients (14.9%) displayed ependymal enhancement. Meningeal enhancement was observed in only in 5 cases (5.7%). Satellite-enhancing lesions were found in 11 cases (12.6%). Only 2 patients showed no edema, whereas all other patients had edema.

### Characteristics of non-enhancing T2-FLAIR hyperintense lesions

**Figure 1:**
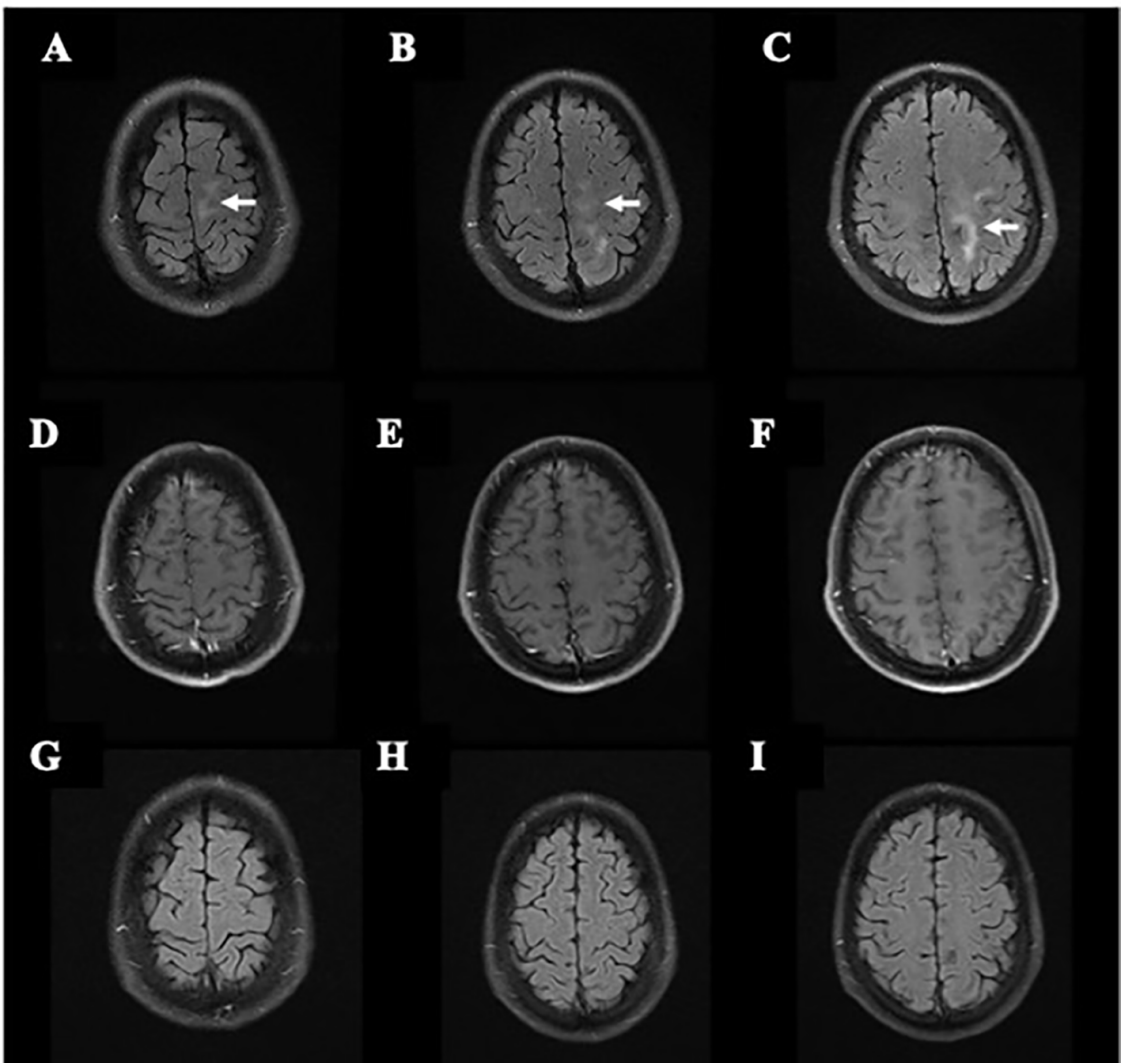
MRI findings in a 43-year-old man. **(A–C)** Axial T2-FLAIR images show patchy, irregular high-signal lesions in the left deep white matter and juxtacortical white matter (white arrow). **(D–F)** These lesions were not enhanced on axial T1-weighted images with contrast enhancement. **(G–I)** After chemotherapy and during the follow-up, axial T2-FLAIR images revealed that the previous high-signal lesions disappeared.

**Figure 2:**
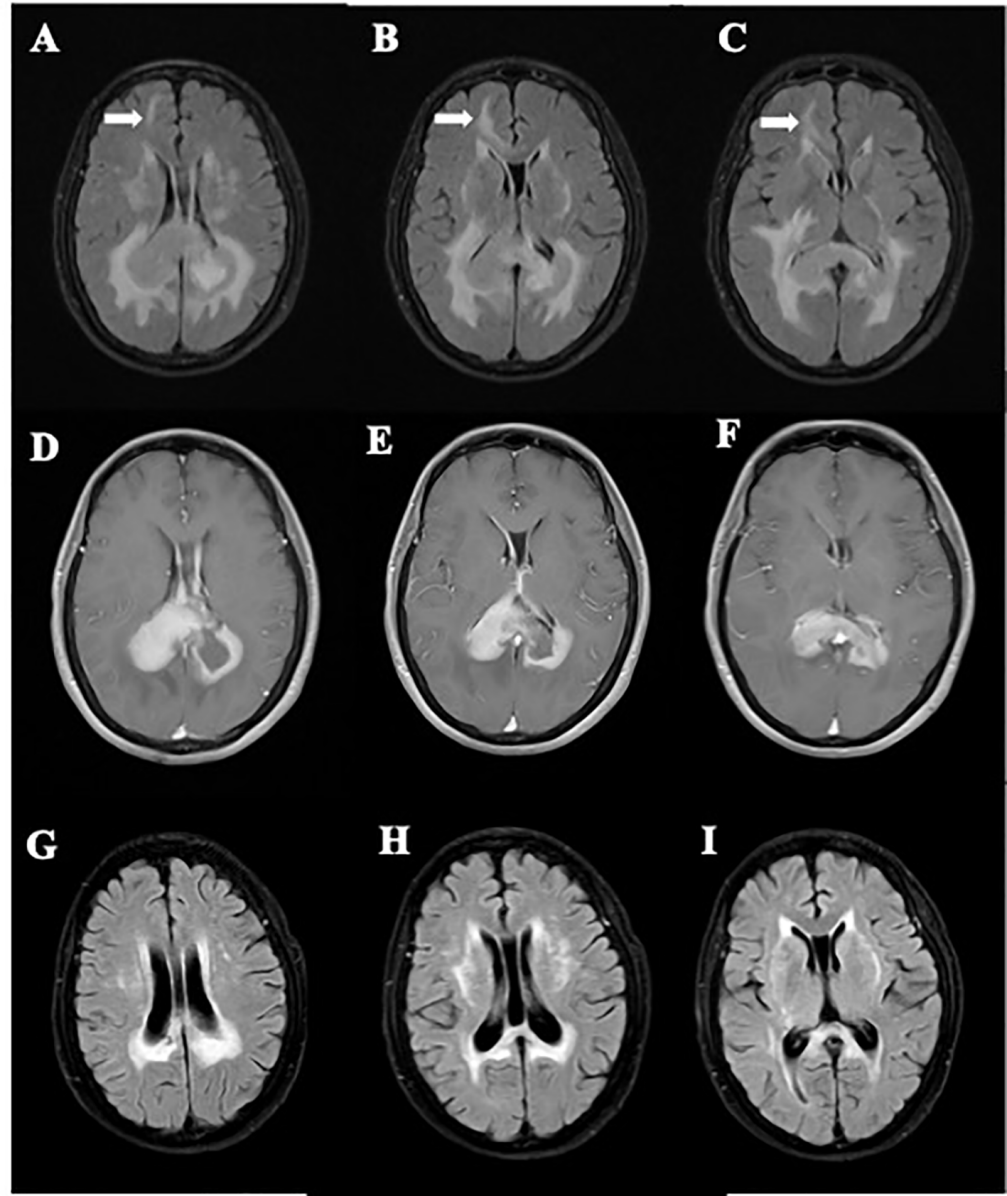
MRI findings in a 51-year-old woman. **(A–C)** T2-FLAIR images show linear high-signal lesions in the deep white matter of the right frontal lobe (white arrow). **(D–F)** These lesions were not enhanced on axial T1-weighted images with contrast enhancement. **(G–I)** After treatment during follow-up, axial T2-FLAIR images revealed that the previous high-signal lesions had markedly decreased.

A total of 10 patients (11.5%) were found to have non-enhancing T2-FLAIR hyperintense lesions located at a distance from the enhancing tumor site at baseline, which disappeared or showed marked reduction after chemotherapy. The median age of these patients was 56.6 years (range: 36–70 years), and 4 (40%) were male. A total of 11 lesions were confirmed in these 10 patients. The median diameter and product of the largest perpendicular diameters of these FLAIR lesions were 23.1mm (range: 12.6–33.9) and 180.1 mm^2^ (range: 91–319.4), respectively. The median T2-FLAIR lesion volume was 1609.3 mm^3^ (range: 809.2–3436.6). A total of 10 lesions disappeared after treatment, and one lesion showed a marked reduction of ≥50% (**Figures 1**, **2**).

Among these lesions, seven lesions were located in the left hemisphere, three lesions in the right hemisphere, and one in the right middle cerebellar peduncle. These non-enhancing lesions were most frequently located in the juxtacortical and deep white matter (n = 7, 63.7%). The other locations included the periventricular white matter (n = 2, 18.2%), basal ganglia (n = 1, 9.1%), and the middle cerebellar peduncle of the infratentorial area (n = 1, 9.1%). These non-enhancing lesions usually had a large volume and irregular shape. For the signal characteristics, all the lesions were hyperintense on T2-weighted images. In contrast, on T1-weighted images, the lesions were hypointense in 5 patients (50%) and isointense in 5 patients (50%). The median ADC value was 0.83 × 10^-^³ mm²/s, with a range of 0.71–1.13 × 10^-^³ mm²/s. Five patients (50%) showed restricted diffusion based on an ADC value < 0.8 × 10^-^³ mm²/s.

### Comparison of baseline demographic and MRI characteristics between patients with and without non-enhancing T2-FLAIR hyperintense lesions

Demographic and baseline MRI characteristics of patients with and without non-enhancing T2-FLAIR hyperintense lesions are presented in **Table 2**. Age and sex showed no significant differences between the two groups. Regarding the number of contrast-enhancing tumor lesions, patients with non-enhancing T2-FLAIR hyperintense lesions exhibited a higher rate of diffuse enhancing lesions (60% vs. 24.7%, *P* = 0.027), compared with the patients without non-enhancing T2-FLAIR hyperintense lesions. In terms of lateralization of enhancing tumor lesions, bilateral involvement was more frequently observed in patients with non-enhancing T2-FLAIR hyperintense lesions than in those without these lesions (70% vs. 19.4%, *P* = 0.011). No significant differences were identified between the two groups with respect to other radiological parameters of the contrast-enhancing tumor lesions, including location and enhancement pattern (*P* > 0.05).

**Table 2:** Comparison of baseline demographic and MRI characteristics between patients with non-enhancing T2-FLAIR hyperintense lesions and patients without non-enhancing T2-FLAIR hyperintensity lesions.

Baseline characteristics	Patients with non-enhancing T2-FLAIR hyperintensity lesions (n = 10)	Patients without non-enhancing T2-FLAIR hyperintensity lesions(n = 77)	P
Age (years)	56.6 (36-70)	54 (16-80)	0.876
Male	4(40.0%)	24(32.9%)	0.728
Number of enhanced lesion(s)			
≤ 2	4	58	0.027*
Multiple (> 2)	6	19	
Lateralization			
Left	1	25	0.011*
Right	0	28	
Bilateral	7	15	
Midline	2	9	
Location			
Deep^#^	7	55	0.583
Superficial	1	14	
Deep and superficial	2	8	
Enhancement type			
Homogeneous	9	61	0.680
Heterogeneous	1	16	

*Mean significant differences.

^#^ Deep lesions include those involving the corpus callosum, basal ganglia, brainstem, and/or cerebellum.

## Discussion

In our study, all PCNSL patients exhibited at least one contrast-enhancing tumor lesion. Meanwhile, we found that 11.5% of patients had non-enhancing T2-FLAIR hyperintense lesions at a distance from the contrast-enhancing tumor site at baseline, which disappeared or markedly decreased after chemotherapy. This dynamic change indicated that these white matter non-enhancing T2-FLAIR hyperintense lesions may have a neoplastic nature. A total of 11 non-enhancing lesions (in 10 patients) were confirmed, among which 10 completely disappeared after treatment and 1 showed a significant reduction. The most frequently involved locations of non-enhancing T2-FLAIR hyperintense lesions were the deep and juxtacortical white matter. When comparing demographics and baseline MRI characteristics between the patients with and without non-enhancing T2-FLAIR hyperintense lesions, we found that the patients with non-enhancing T2-FLAIR hyperintense lesions exhibited a higher rate of bilateral lesions and diffuse enhancing lesions (p < 0.05).

Radiologic features of newly diagnosed PCNSL in immunocompetent patients have been described comprehensively (12–14). Gadolinium-enhanced MRI is the main imaging modality for the initial work-up of PCNSL and for response assessment after chemotherapy. The majority of the PCNSL patients in our study exhibited typical deeply located contrast-enhancing lesions. The most frequently involved areas were the cerebral hemisphere, basal ganglia, and thalamus. These findings were consistent with previous studies. However, non-enhancing lesions in PCNSL patients, which were subsequently proven to be B-cell lymphoma, have been reported occasionally (12, 14, 18, 20). It has been reported that these non-enhancing tumor lesions could present either as a component or as the sole radiological finding in PCNSL patients (15, 17, 18, 21). Some of those cases exhibited diffuse brain infiltration with lymphoma cells, termed lymphomatosis cerebri, which accounts for approximately 1% of PCNSL cases (22, 23). Intravascular lymphoma, a rare variant of aggressive (usually B-cell) non-Hodgkin lymphoma characterized by selective growth of neoplastic cells within blood vessel lumina, is another commonly reported subtype of non-enhancing PCNSL (24). Diagnostic delay is a common problem in PCNSL, not only in cases with complete absence of enhancement but also in patients with contrast-enhancing tumor lesions.

In fact, when non-enhancing lesions present together with enhancing tumor lesions in PCNSL, they may be overlooked or interpreted as non-tumor lesions according to the current response criteria of the International Primary CNS Lymphoma Collaborative Group (IPCG) (25). Furthermore, a brain biopsy is usually not performed at these non-enhancing sites. To date, the significance of non-enhancing T2-FLAIR hyperintense lesions in PCNSL remains unclear. Tabouret et al. first reported the phenomenon that some non-enhancing T2-FLAIR hyperintense lesions distant from the enhancing tumor site in PCNSL patients were found to be markedly decreased after chemotherapy, which is in line with our study (18). In our study, the majority of these lesions completely disappeared after chemotherapy. This marked reduction or complete disappearance of these non-enhancing T2-FLAIR hyperintense lesions strongly supports their neoplastic nature. Furthermore, in the study by Tabouret et al., 27.8% of patients who had non-enhancing T2-FLAIR hyperintense lesions with dynamic change relapsed in the initially non-enhancing T2-FLAIR lesions. This further indicated that these non-enhancing T2-FLAIR hyperintense lesions were probably neoplastic lesions. Several previous studies have reported that non-enhancing lesions were proven to be lymphoma tissue through biopsy or postmortem examination (14, 17). Moreover, tumors can be identified in regions with completely normal radiological findings, even on T2-weighted images and T2-FLAIR images (17). In our study, we found that the patients with chemotherapy-responsive non-enhancing T2-FLAIR hyperintense lesions were more likely to have diffuse and bilateral enhancing tumor lesions. Collectively, these findings suggest that PCNSL is, in fact, a whole-brain disease and that enhancing lesions on MRI do not necessarily correlate with the true extent of the tumor (26).

Our study found that patients with more diffusely infiltrating lymphoma were prone to have chemotherapy-responsive non-enhancing T2-FLAIR lesions, which may represent the early-stage tumor tissue. Pathology reveals that PCNSL consists of highly proliferative tumor cells growing in an angiocentric growth pattern, in which lymphoma cells accumulate around small- and medium-sized blood vessels (27). During the angiotropic invasive growth of the tumor, tumor cells arrange themselves centripetally around the Virchow–Robin space and show a sleeve-like infiltration pattern. As these lesions grow larger, disruption of the blood-brain barrier occurs, enabling their detection via contrast enhancement. Routine contrast-enhanced MRI only reflects the disruption of the blood-brain barrier rather than the degree of tumor angiogenesis. Thus, early-stage tumor lesions that have not yet disrupted the blood-brain barrier may not show contrast enhancement at all. Previous studies have reported several PCNSL cases that initially presented as non-enhancing CNS lymphoma but subsequently showed enhancement with disease progression (15, 17). DeAngelis et al. demonstrated that tumor cells may reside behind a relatively intact blood-brain barrier on autopsy (17). Therefore, we speculate that the chemotherapy-responsive non-enhancing T2-FLAIR lesions likely represent the early phase of lymphoma, which has not yet disrupted the blood-brain barrier. It is important to recognize that contrast enhancement is a surrogate for disruption of the blood-brain barrier and may not accurately reflect the true extent of the tumor in PCNSL patients.

Although it has been widely accepted that tumor lesions in PCNSL patients could present without enhancement, it is still very challenging to distinguish these non-enhancing tumor lesions from non-specific white matter hyperintense lesions, which can be caused by diverse etiologies, including migraine, stroke, dementia, and healthy aging (28–30). In our study, we observed that the majority of the potentially neoplastic non-enhancing T2-FLAIR hyperintense lesions were located in the deep and juxtacortical white matter and were characterized by relatively large volumes and irregular shapes. Meanwhile, we found that 60% of patients with chemotherapy-responsive non-enhancing T2-FLAIR lesions showed restricted diffusion of these non-enhancing lesions. It has also been reported that PCNSL lesions exhibit lower ADC values (median 0.71 × 10^-^³ mm²/s), including one case of non-enhancing PCNSL, reflecting their higher cellularity, regardless of whether restricted diffusion was present (10). The authors suggest that quantitative measurement of ADC values is an important adjunct to anatomical imaging in patients with atypical PCNSL. Since the chemotherapy-responsive non-enhancing lesions in our study were not initially considered as tumors, advanced imaging modalities, such as MR spectroscopy and fiuorodeoxyglucose (FDG)-positron emission tomography (PET), were not performed or analyzed. The metabolic characteristics of these chemotherapy-responsive non-enhancing T2-FLAIR lesions were unknown. We speculate that these potentially neoplastic non-enhancing lesions may exhibit a choline peak and regional hypermetabolism on MR spectroscopy and FDG-PET, findings that would differ from those of vascular lesions. In patients with non-enhancing PCNSL, regional hypermetabolism has been identified in areas with MRI abnormalities, which is useful for differentiating non-enhancing PCNSL from other non-specific white matter abnormalities, such as Binswanger disease (31, 32). Meanwhile, vascular lesions associated with cerebral small vessel disease often are accompanied by other well-known imaging markers, including enlarged perivascular spaces, lacunes, and cerebral microbleeds (33). All these characteristics, including ADC value and metabolic features, might be helpful for differentiating non-enhancing white matter high signal lesions of suspicious tumor nature from other diseases. Future studies should investigate quantitative measurements of ADC values, MR spectroscopy, and FDG-PET as complementary tools to explore the metabolic features and biochemical changes of these chemotherapy-responsive non-enhancing lesions and to contribute to optimized evaluation of tumor burden.

More importantly, understanding the characteristics of non-enhancing T2-FLAIR hyperintense lesions of neoplastic nature is key for accurate evaluation of tumor burden. This may provide valuable insights into the mechanisms of PCNSL pathogenesis and contribute to the development of more appropriate treatment strategies. It is generally recognized that the radiographic appearance of the tumor underestimates the true extent of disease (17, 18, 26). However, non-enhancing lesions are not currently incorporated into the current IPCG response criteria (25, 34). All types of response, including complete response, complete response unconfirmed, partial response, stable disease, progressive disease, and relapse, are defined according to the changes in contrast-enhancing lesions. For example, according to the IPCG response criteria, the absence of contrast-enhancing lesions in the absence of other lymphoma manifestations is considered a complete response in PCNSL. It has been reported that the extent of radiological response based solely on contrast-enhancing tumor lesions does not correlate well with survival outcomes in PCNSL (34). This strongly indicates that non-enhancing tumor lesions should be incorporated into refined criteria defining response and progression in PCNSL. A recent study reported that non-enhancing PCNSL may be less aggressive than enhancing tumors (35).

Our study has several limitations. First, it was an observational, retrospective cohort study, which may introduce some biases due to confounding factors. Second, the sample size of our study was relatively small. These findings need to be further validated in a larger longitudinal cohort.

## Conclusion

The present study found that 11.4% of patients with PCNSL displayed chemotherapy-responsive non-contrast-enhancing T2-FLAIR hyperintense lesions at a distance from the enhancing tumor lesions, which strongly indicates their neoplastic nature. Those lesions should be incorporated into initial diagnosis of PCNSL. Further studies should be performed to investigate the incidence, metabolic features, and biochemical changes of non-enhancing PCNSL lesions in larger cohorts to better understand the characteristics of non-contrast enhancing T2 FLAIR hyperintense lesions with potential neoplastic nature.

## Data Availability

The original contributions presented in the study are included in the article/supplementary material. Further inquiries can be directed to the corresponding author.
